# Artificial and biological supports are different for pea plants

**DOI:** 10.1080/15592324.2024.2355739

**Published:** 2024-06-05

**Authors:** Bianca Bonato, Valentina Simonetti, Silvia Guerra, Umberto Castiello

**Affiliations:** Department of General Psychology, University of Padova, Padova, Italy

**Keywords:** Pea plants, kinematics, accuracy, climbing plants, circumnutations, plant behavior, plant movement

## Abstract

Previous studies on the kinematics of pea plants’ ascent and attach behavior have demonstrated that the signature of their movement varies depending on the kind of support. So far, these studies have been confined to artificial supports (e.g. wooden sticks). Little is known regarding the conditions under which pea plants could rely on biological supports (e.g. neighboring plants) for climbing toward the light. In this study, we capitalize on the 3D kinematic analysis of movement to ascertain whether pea plants scale their kinematics differently depending on whether they aim for artificial or biological support. Results suggest that biological support determines a smoother and more accurate behavior than that elicited by the artificial one. These results shed light on pea plants’ ability to detect and classify the properties of objects and implement a movement plan attuned to the very nature of the support. We contend that such differences depend on the augmented multisensory experience elicited by the biological support.

## 1 Introduction

Climbing plants need external support to maximize light absorption and develop vertically. Climbing plants have evolved several morphological tricks to clasp support, with the development of tendrils being the most highly sophisticated. Tendrils are modified leaves or flower peduncles sensitive to mechanical stimulation and capable of coiling around potential support.^1^ Tendrils show helical movements (i.e., circumnutation) to explore the environment, and once potential support is perceived, they orient their movement toward it.

Among tendril – bearing plants, pea plants (*Pisum sativum L*. from now on *P. sativum*) are the most studied at the genetic, morphological, physiological, and behavioral levels.^2–9^ Recent studies have capitalized on timelapse techniques to investigate the kinematical features of their movement when aiming at potential supports with different characteristics. Guerra et al.^3^ focused their analysis on support thickness. They examined a condition lacking potential support and one in which a support of a different thickness (i.e., thin or thick) was nearby. The results showed that when the plants perceived the presence of the support, they rapidly changed the direction of their circumnutating movement to approach and grasp it. The plants adjusted the kinematics of their coming and grasping movement in terms of their tendrils’ velocity and aperture (i.e., the maximum distance between the tips of the tendrils) depending on support thicknesses. Plants moved more quickly and opened their tendrils more in the presence of a thinner support.

One may ask why climbing plants should vary their kinematic patterning depending on the thickness of the support. A reasonable hypothesis is that the metabolic cost of morphological modulation and circumnutations may vary.^10,11^ Studies on different varieties of climbers suggest that if the size (diameter) of potential supports increases beyond some point, climbing plants are unable to maintain tensional forces and, therefore, lose attachment to the support.^12–15^ The fact that pea plants decrease their average and maximum tendril velocity in the presence of thick support may allow for acquiring more information about the thick support, which is considered a more demanding task, and implementing corrective adjustments to reduce the possible risk of errors. The reduced velocity may permit the plant to save energy and invest in the modulation and correction of the trajectories for a more accurate selection of the contact points to twine around the support.

Accuracy appears to be an essential aspect of climbers’ behavior. It is operationalized through corrective adjustments (i.e., submovements), which reduce any spatial discrepancy between the effector and target position.^16^ This principle is also applicable to P. sativum plants. As recently demonstrated, they produce more submovements in the presence of thicker than thin-ner supports, confirming that climbers found thicker supports more demanding.^17^

So far, these foundational studies concerning how pea plants scale their kinematics depending on their aim have been confined to inanimate supports (e.g., wooden sticks). Little is known regarding how they could rely on neighboring plants to climb toward the light. This condition can be observed in numerous climbing plants in open environments.^18,19^ When potential support is not readily available, climbing shoots twine around another plant, providing biological support.^7,19^

In the present study, we address this issue by asking whether there might be a difference in the kinematics of pea plants’ ascent and attach movements depending on the nature of the support they aim for. Differently from aiming at static support, aiming toward another plant implies keeping into account a variety of cues ranging from morphological (e.g., the size of the stem) to tactile (e.g., the information coming from the two plants touching parts) to chemical (e.g., volatile organic compounds), and motion (e.g., the movement of the hosting plant). A multisensory experience may have implications when establishing how to interact with biological support. If the biological support triggers a sensorial crosstalk between the agent and the support plant, we expect a smoother and more accurate behavior than the static/artificial support elicits. This should be revealed through kinematical analysis concerning accuracy measurements, namely the number of sub-movements and the end-point variability.

## 2 Materials and methods

### 2.1 Subjects

Sixteen snow peas (*Pisum sativum* var. *saccharatum* cv Carouby de Maussane) were chosen as the study plants. Pea seeds were selected, potted, and kept at the conditions outlined below. We determined our sample size based on previous studies e.g.^17^

### 2.2 Experimental conditions

**Figure 1:**
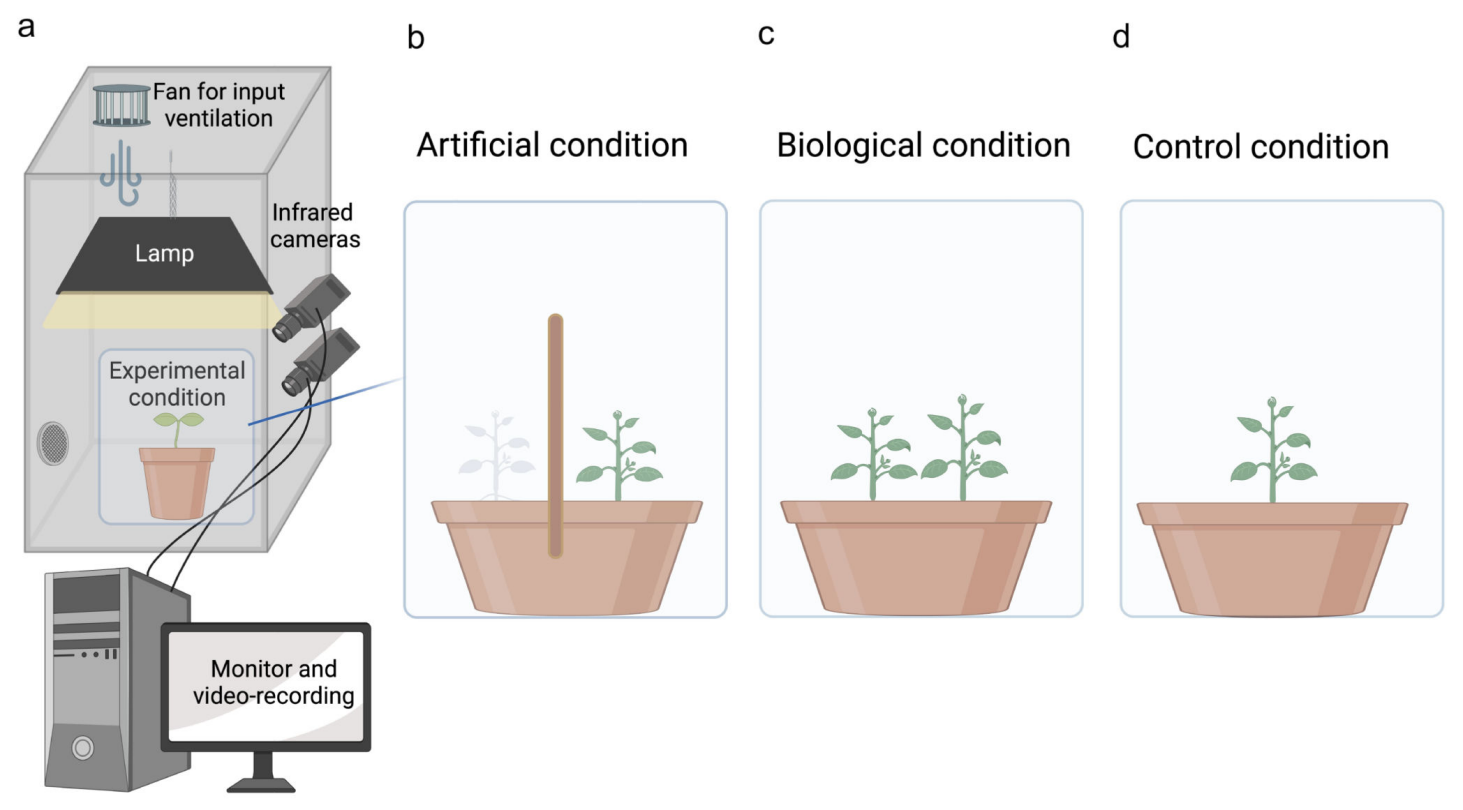
In panel (a): Graphical representation of the experimental setup. A growth chamber with a controlled environment in terms of light and temperature and two infrared cameras recording the movement of the plants 24 hours per day. In panel (b): graphical representation of the experimental condition for the artificial support (AC condition). In panel (c): graphical representation of the experimental condition for the biological support (BC condition). In panel (d): graphical representation for the control condition (CC condition).

For the “biological” support condition (BC), pea plants were potted in the presence of another plant (see supplementary video BC_condition). In such circumstances, the plants were somewhat constrained to intertwine with the other plants to climb toward the light. The plant considered for the analysis was the one that finalized the movement, grasping the other plant at the level of the stem to ensure a firm attachment. For the “artificial” support condition (AC), pea plants grew in the presence of an inanimate wooden pole (see supplementary video AC_condition). The wooden pole was 60 cm in height and 1.2 cm in diameter (Figure 1). The in-ground part of the stimulus was 7 cm, while the above-ground part was 53 cm. The pole was 12 cm from the plant’s first unifoliate leaf. To equate the BC condition, we introduce another plant positioned 12 cm from the wooden pole (Figure 1). Note that this plant did not touch the wooden pole. In other words, it was a passive presence to counterbalance the number of plants considered for the BC condition. Note that despite the presence of two plants, only one plant was at a reachable distance and, therefore, in the position to grasp the support. A control condition (CC) was also considered in which an individual plant grew without support (see supplementary video CC_condition).

### 2.3 Dependent measures

The dependent variables specifically tailored to test our experimental hypothesis were chosen based on previous kinematic studies in pea plants^6,7,10,17,20^ and the literature concerning submovements and the fine structure of movement.^17^ If we zoom in on the fine structure of movement, we observe a primary movement and a series of submovements that perfect the homing phase. An easy way to recognize submovements is by considering how often the velocity waveform crosses the zero axis (i.e., submovement_velocity). The same applies to the acceleration profile (i.e., submovement_acceleration) and the jerk profile (i.e., submovement_jerk). These measures were: (i) the total duration of the tendrils’ circumnutations; (ii) the amplitude of mean velocity reached by the tendrils; (iii) the number of submovements_velocity computed as the total number of zero crossing from the velocity profile performed in the last 10% of the movement time; (iv) the number of submovements_acceleration computed as the total number of zero crossing from the acceleration profile performed in the last 10% of the movement time; (v) the number of submovements_jerk computed as the total number of zero crossing from the jerk profile performed in the last 10% of the movement time; (vi) the total number of submovements (acceleration, velocity and jerk) in the last 10% of movement time; (vii) the variability of the endpoint positions at the end of the movement.

### 2.4 Data analysis

In order to quantify the *submovement_velocity*, we defined the following parameter concerning velocity array computation in a discrete mood:

$${\text{v}}_{i}=\frac{{\text{s}}_{i}-{\text{s}}_{\left(i-1\right)}}{{\text{t}}_{i}-{\text{t}}_{\left(\text{i}-1\right)}}$$

Where ***v***_***i***_ is the velocity computed at time point i, ***s***_***i***_ is distance from the stimulus on the XZ plane at time point i and ***t***_***i***_ is the time for point i referring to the last 10% of the movement.

To quantify the *submovement_acceleration*, we defined the following parameter concerning acceleration array in a discrete mood as a derivative of the velocity previously described:

$${\text{a}}_{i}=\frac{{\text{v}}_{i}-{\text{v}}_{\left(\text{i}-1\right)}}{{\text{t}}_{i}-{\text{t}}_{\left(\text{i}-1\right)}}$$

Where ***a***_***i***_ is the acceleration computed at time point i, ***v***_***i***_ is the velocity computed at time point i and ***t***_***i***_ is the time for point i referring to the last 10% of the movement.

In order to quantify the *submovement_jerk*, we defined the following parameter concerning jerk array in a discrete mood as the rate of changes of the acceleration previously described:

$${\text{j}}_{i}=\frac{{\text{a}}_{i}-{\text{a}}_{\left(\text{i}-1\right)}}{{\text{t}}_{i}-{\text{t}}_{\left(\text{i}-1\right)}}$$

Where ***j***_***i***_ is the jerk computed at time point i, ***a***_***i***_ the acceleration computed at time point i and ***t***_***i***_ as the time for point i referring to the last 10% of the movement.

The analysis used JASP^21^ nested within the environment R;^22^ see used packages: https://jasp-stats.org/r-package-list/). We performed the Shapiro-Wilk test to check the normality of the dataset. Since the data were not normally distributed, we conducted a nonparametric Kruskal-Wallis Test to compare the different conditions’ movement duration and mean velocity. A Tukey’s post-hoc comparison has been performed. A parametric Student t-test was conducted to compare the dependent measures concerned with the submovements between the AC and the BC conditions. No analysis of submovements was possible for the CC condition because there was no grasping phase.

## 3 Results

### 3.1 Qualitative results

**Figure 2:**
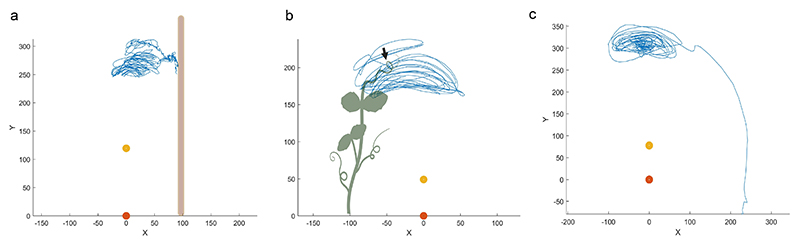
Example of trajectories for the AC (a), the BC (b), and the CC (c) condition. The yellow and orange dots represent the stem of the tracked plant. The blue line represents the trajectory of the tendril. The black arrow in Panel (b) represents the end of the movement for the BC, culminating with the final grasping phase on the support plant.

All plants exhibited a growing pattern characterized by circumnutation (Figure 2a–c). By observing the trajectories, we can see a different pattern for the two experimental conditions. AC is characterized by a higher density of circumnutation and smaller movements in the very proximity of the support than BC. This may allow for implementing more corrective adjustments to reduce the possible risk of errors. This careful approach may permit plants to save energy and invest in the modulation and correction of the trajectories for a more accurate selection of the contact points. For BC, circumnutations appear smoother, and the other plant is grasped with a single final maneuver, as indicated by the black arrow. There are no modulations or online corrections of the trajectories during the final phase. This suggests an easier and sharper movement concerning the one performed toward the artificial support. For the CC condition, the circumnutative movement ends with an irreversible plant drop represented by the linear trajectory directed toward the floor (Figure 2c).

### 3.2 Kinematic results

The duration of the circumnutation is shorter, and the mean velocity is faster for the AC condition than the BC condition (see Tables 1–3), suggesting a more careful approach when the support is biological. The plants perform a different movement when there is no support in the surroundings.

**Figure 3:**
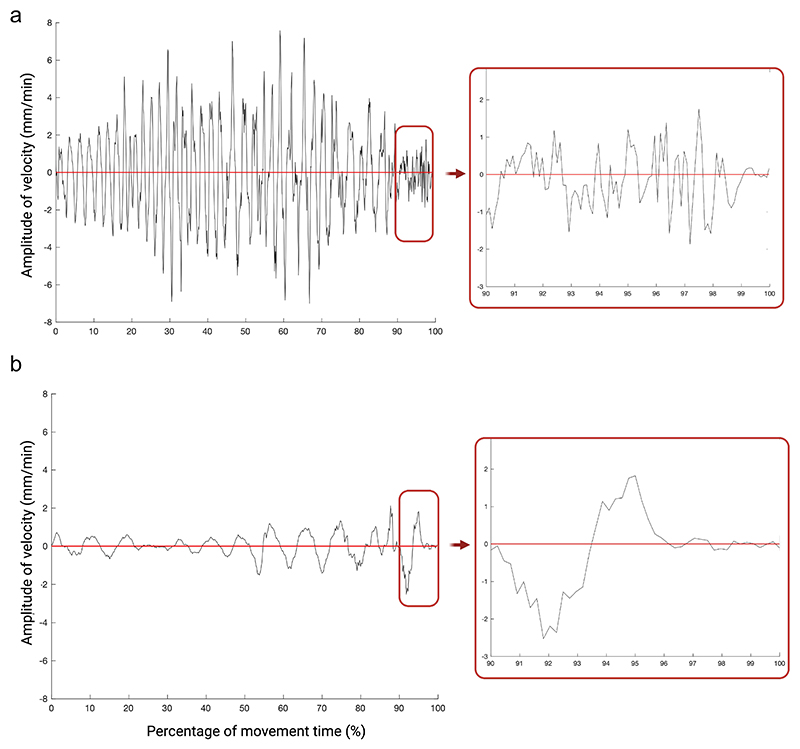
Graphical representation of the velocity profile for exemplar plants for the two conditions. The callouts represent the last 10% of the movement, emphasizing the number of zero-crossings on the velocity profile representing the amount of type 1 submovements for the AC condition (a) and BC condition (b). Note that the number of submovements is much higher for the AC condition than for the BC condition.

The number of submovements is higher for the AC than for the BC condition (see Tables 4 and 5). This is particularly evident in the final 10% of the movement (Figure 3).

For endpoint variability, this value for the AC condition (DS = 45.776) is more significant than for the BC condition (DS = 11.294) (see Table 4), suggesting that greater accuracy in determining the contact points on the wooden pole is needed to accomplish the final grasping.

## 4 Discussion

The present study aimed to investigate whether and how pea plants can differentiate, adjust, and ultimately regulate their ascent and attachment behavior according to the nature of potential support, namely biological (BC condition) or artificial (AC condition). To do this, we capitalized on the concept of accuracy, as exemplified via the occurrence of sub-movements and the measurement of end-point variability in plants.^17^

According to the findings, pea plants can categorize the type of support they aim for and act accordingly. Movement duration was shorter, and the velocity peak was higher for the AC condition than for the BC condition. Further, more sub-movements and a higher endpoint variability for the AC condition were found. Overall, this kinematical patterning indicates that the movement toward a conspecific is slower and characterized by a more accurate homing phase.

Given the absence of studies in the plant’s realm concerned with the issues at stake, it is tempting to discuss this aspect with a comparative and integrated approach.^23,24^ Animal research revealed that the kinematics of an agent’s movement during the interaction with a biological agent is different from that underlying the identical movement implying the interaction with an inanimate object,^25,26^ with the former characterized by a more accurate patterning mirroring the one described here for pea plants. The fact that our plants act toward a conspecific implies greater accuracy, which becomes particularly evident when looking at the differences in sub-movements and endpoint variability. For the BC condition, the observed number of sub-movements and the endpoint variability are lower than for the AC condition. One may say that it is somewhat counterintuitive that support still requires more adjustments than one that moves, and a more complex structure characterizes it. The greater smoothness of the movement associated with the BC condition could rely on exchanging information between the two plants at both roots and aerial levels. This may occur via a continuously updated interaction through volatile compounds. Plants use one of the most efficient and complex mechanisms to communicate volatile organic compounds (VOCs). A chemical “language” plants use for a variety of reasons, including exchanging useful information,^27–29^ adapting to environmental stress^30^ and mediating plant interactions^31^ with other organisms both above and below ground. Underground, this chemical communication occurs through root exudates that convey crucial information about the neighbors in the surrounding environment.^32,33^ Furthermore, it cannot be excluded that they may be able to discriminate detailed features of what surrounds them via a primitive visual system.^34^ Evidence suggests that the leaf upper and sub-epidermis comprise cells suitable to act as ocelli, allowing plants to experience a sort of vision.^34,35^ Support for this contention comes from studies on leaf mimicry in climbing plants^36^ and photoreceptor-mediated kin recognition of Arabidopsis seedlings.^37^ Both reports suggest plants can gather information about their environmental setting through vision-based inputs and behave accordingly.

In light of this, again, comparative literature might be of help. Several studies have been conducted on other species as primates,^38,39^ mammalian,^40^ and insects,^41–45^ describe how the integration of complementary information exchange affords numerous perceptual and behavioral benefits increasing accuracy^46–48^ and resulting in a smoother behavior.

Considering all the information, it seems that there are things in the world with which plants interact more smoothly than others. Identifying them is fundamental for plants. How do plants identify objects and separate conspecifics from all the rest? It seems that they can track and individuate the two things differently and interact appropriately with them, a method of identification that might be termed a property method. In this broad classification, members’ conspecifics are particularly important. Various properties may allow plants to uniquely identify conspecifics and segregate them from everything else, such as having a particular morphological schema or speaking a natural chemical ‘language.’ It is reasonable to suppose that plants can discriminate a wide array of properties not limited to basic low-level stimulus features but rather extend to the complex properties that could allow them to single out conspecifics uniquely. Further, such abilities might not just be “perceptual.” Not only can they discriminate other plants from artifacts, as it seems here, but they can also form specific expectations of what they can do with them and behave accordingly. Plants seem equipped with all the necessary mechanisms for applying the proposed ‘property method,’ at least to plants and objects. Nonetheless, we do not know whether they use the concepts and property detectors to identify objects. This study has been the first step in shedding light on the highly controlled and accurate mechanisms plants put into work when acting toward different kinds of supports with other properties. We now know plants are good at detecting property changes and planning the movement and accuracy accordingly. What remains to be uncovered is what the metaphysics of this kind of object identification could look like.

## Figures and Tables

**Table 1:** Descriptive statistics for the kinematic measures when comparing the AC and the BC conditions.

		Median	IQR	P-value of Shapiro-Wilk	Range	25th percentile	50th percentile	75th percentile
Total duration movement	BC	111.000	39.000	.857	144.000	93.000	111.000	132.000
	AC	81.000	30.000	<.001	120.000	69.000	81.000	99.000
	CC	63.000	18.000	<.001	159.000	57.000	63.000	75.000
Mean velocity	BC	1.582	1.694	<.001	4.275	0.714	1.582	2.408
	AC	2.571	1.956	<.001	7.060	1.564	2.571	3.520
	CC	2.172	2.839	<.001	6.484	1.174	2.172	4.013

**Table 2:** Kruskal-Wallis non parametric ANOVA among AC, BC and control condition for the dependent measures considered.

Cases	Statistic	df	p
Total duration	146962.276	2	<.001
Mean speed	42.620	2	<.001

Kruskal-Wallis Test.

**Table 3:** Post-hoc comparison for the dependent measures considered.

Total duration		Mean Difference	SE	t	p_tukey_
CC	BC	−43.218	0.166	6.302	<.001
	AC	−18.192	0.131	0.210	0.976
BC	AC	−1.020	0.172	−5.941	<.001
Mean velocity					
CC	BC	1.047	0.166	6.302	<.001
	AC	0.027	0.131	0.210	0.976
BC	AC	−1.020	0.172	−5.941	<.001

P-value adjusted for comparing a family of 3.

**Table 4:** Descriptive statistics for the specific dependent measures considered in terms of sub-movements for the AC and the BC conditions.

		Mean	Sth. Deviation	Shapiro-Wilk	P-value of Shapiro-Wilk	Minimum	Maximum
Submovement_type1	BC	48.000	13.148	0.884	.207	33.000	66.000
	AC	67.125	20.490	0.991	.997	35.000	101.000
Submovement_type2	BC	26.250	7.025	0.961	.817	14.000	35.000
Submovement_type3	AC	43.250	21.684	0.948	.688	17.000	81.000
	BC	37.500	7.982	0.887	.220	26.000	46.000
Total submovement	AC	59.375	26.403	0.969	.891	24.000	100.000
	BC	115.500	23.513	0.951	.722	79.000	145.000
	AC	169.750	63.209	0.951	.722	80.000	259.000
End point variability	BC	18.072	11.294	0.977	.949	0.369	34.438
	AC	242.397	45.776	0.973	.919	175.622	307.567

**Table 5:** Independent samples student’s T-Test between AC and BC condition for the dependent measures considered.

						95% CI for Cohen’s d	
	t	df	P	Cohen’s d	SE Cohen’s d	Lower	Upper
Submovement_type1	−2.222	14	.043*	−1.111	0.572	−2.156	−0.033
Submovement_type2	−2.109	14	.053	−1.055	0.565	−2.092	0.015
Submovement_type3	−2.243	14	.042*	−1.122	0.573	−2.168	−0.042
Total submovements	−2.275	14	.039*	−1.138	0.575	−2.186	−0.055

Student’s t-test.

## Data Availability

The data supporting this study’s findings are available in ZENODO at https://doi.org/10.5281/zenodo.10852877.
